# Tooth serration morphologies in the genus *Machimosaurus* (Crocodylomorpha, Thalattosuchia) from the Late Jurassic of Europe

**DOI:** 10.1098/rsos.140269

**Published:** 2014-11-05

**Authors:** Mark T. Young, Lorna Steel, Stephen L. Brusatte, Davide Foffa, Yves Lepage

**Affiliations:** 1 School of GeoSciences, Grant Institute, University of Edinburgh, The King's Buildings, Edinburgh EH9 3FE, UK; 2 School of Ocean and Earth Science, National Oceanography Centre, University of Southampton, Southampton SO14 3ZH, UK; 3 Department of Earth Sciences, Natural History Museum, London SW7 5BD, UK; 4 National Museums Scotland, Chambers Street, Edinburgh EH1 1JF, UK; 5 Sciences et Géologie Normandes, 76620 Le Havre, France

**Keywords:** dental morphology, enamel ridges, Europe, *Machimosaurus*, ‘pseudo-denticles’, Teleosauridae

## Abstract

*Machimosaurus* was a large-bodied durophagous/chelonivorous genus of teleosaurid crocodylomorph that lived in shallow marine and brackish ecosystems during the Late Jurassic. Among teleosaurids, *Machimosaurus* and its sister taxon ‘*Steneosaurus’* *obtusidens* are characterized by having foreshortened rostra, proportionally enlarged supratemporal fenestrae and blunt teeth with numerous apicobasal ridges and a shorter anastomosed ridged pattern in the apical region. A recent study on ‘*S*.’ *obtusidens* dentition found both true denticles and false serrations (enamel ridges which contact the carinae). Here, we comprehensively describe and figure the dentition of *Machimosaurus*, and find that *Machimosaurus* *buffetauti* and *Machimosaurus hugii* have four types of serration or serration-like structures, including both denticles and false denticles on the carinae. The denticles are irregularly shaped and are not always discrete units, whereas the false denticles caused by the interaction between the superficial enamel ridges and the carinae are restricted to the apical region. Peculiarly, the most ‘denticle-like’ structures are discrete, bulbous units on the apicobasal and apical anastomosed ridges of *M. hugii*. These ‘pseudo-denticles’ have never, to our knowledge, previously been reported among crocodylomorphs, and their precise function is unclear. They may have increased the surface area of the apical region and/or strengthened the enamel, both of which would have been advantageous for a durophagous taxon feeding on hard objects such as turtles.

## 2. Introduction

Teleosaurids were an evolutionary radiation of crocodylomorphs commonly found in coastal, lagoonal and brackish ecosystems during the Jurassic [1–4]. Within Teleosauridae, there is a Middle–Late Jurassic ‘*Steneosaurus*’ *obtusidens*–*Machimosaurus* subclade [5,6], which is often considered to be durophagous/chelonivorous owing to a suite of craniodental morphologies that would have been well suited for feeding on hard-shelled turtles or thick-scaled fishes, i.e. a foreshortened snout, proportionally enlarged supratemporal fenestrae and blunt, heavily ornamented dentition [3,7–12]. However, recently a generalized ‘macrophagous’ diet has been posited for ‘*S.*’ *obtusidens* based on tooth morphology, particularly the presence of serrations [13].

Scanning electron microscopy (SEM) and macrophotographic investigation of ‘*S*.’ *obtusidens* dentition find two types of tooth serrations: false denticles and true denticles [13]. False denticles are defined as being formed by the superficial enamel contacting the carinal keel, as often occurs when enamel ornaments such as ridges intersect with the carina [14]. True denticles are defined following Young *et al*. [13]: ‘true serrations are discrete morphological units on, or along, the carinae which are not formed by the surface enamel ornamentation’. (Note that we do not use a histological definition of denticles, namely that the underlying dentine contributes to them, owing to the destructive nature of the analyses needed to confirm this.) These discrete morphological units may, or may not, be clearly individualized by interdenticular grooves or notches. In certain cases, there is no interdenticular groove, because there is a high carinal keel and/or the denticles are microscopic and poorly defined. This occurs in taxa with incipient denticles, such as in ‘*S*.’ *obtusidens* [13] and the metriorhynchid thalattosuchians ‘*Metriorhynchus*’ *brachyrhynchus*, *Tyrannoneustes lythrodectikos* and *Torvoneustes carpenteri* [15,16]. The definition used herein is a revised version of that used by Prasad & Broin [14], as incipient denticles are not always clearly individualized, and may not form a contiguous series.

Serrations are common in taxa with carnivorous or piscivorous diets, ranging from theropod dinosaurs and many Mesozoic marine reptiles to monitor lizards and sabre-toothed cats. They are apparently rare, however, within Teleosauridae. In addition to the ‘*S.*’ *obtusidens*holotype, the only other teleosaurid specimen described as having serrated dentition is the neotype of *Machimosaurus mosae*, which Prasad & Broin [14] considered to have crenulated carinae with false denticles (note that here we use the revised *Machimosaurus* taxonomy of Young *et al*. [4]). Unfortunately, the *M. mosae* neotype is no longer in a public collection, and is believed to reside in a private collection; thus, we could not investigate its dental morphologies. With that said, the *M*. *mosae* neotype is only one of numerous *Machimosaurus* specimens from the Late Jurassic of Europe. The dental morphologies of these specimens have yet to be comprehensively studied, so it is currently unclear whether serrations may be more widespread in this characteristic and wide-ranging marine crocodylomorph, and if so, which serration types may be present in which taxa and what implications they may have for understanding feeding ecology.

Here, we investigate the surface morphology of *Machimosaurus* dentition. We identify and describe four distinct serration, and serration-like, morphologies: (i) an incipient denticle morphology very similar to that seen in ‘*S.*’ *obtusidens*, (ii) false denticles created by the enamel ridges contacting the carinal keel (i.e. the carinae), (iii) apicobasal and anastomosed enamel ridges that are ‘wavy’/undulating and create a serration-like effect, and (iv) in *Machimosaurus hugii* numerous apical anastomosed ridges that have discrete units which would be called denticles if they were on the carinae. This final serration-like morphology is intriguing, and to the best of our knowledge has never previously been described for marine crocodylomorph teeth, although it is similar to enamel surface features seen in some other groups of durophagous vertebrates.

### 2.1 Institutional abbreviations

DFMMh, Dinosaurier-Freilichtmuseum Münchehagen, Lower Saxony, Germany; MG, Museu Geológico, Lisbon, Portugal; MPV, Musée paléontologique (Paléospace) de Villers-sur-Mer, Normandy, France; MNHN, Muséum national d’histoire naturelle, Paris, France; NHMUK, Natural History Museum, London, United Kingdom; NMS, Naturmuseum Solothurn, Switzerland; SMNS, Staatliches Museum für Naturkunde Stuttgart, Germany.

## 3. Dental description

### 3.1 Tooth morphology of *Machimosaurus* species

**Figure 1.**
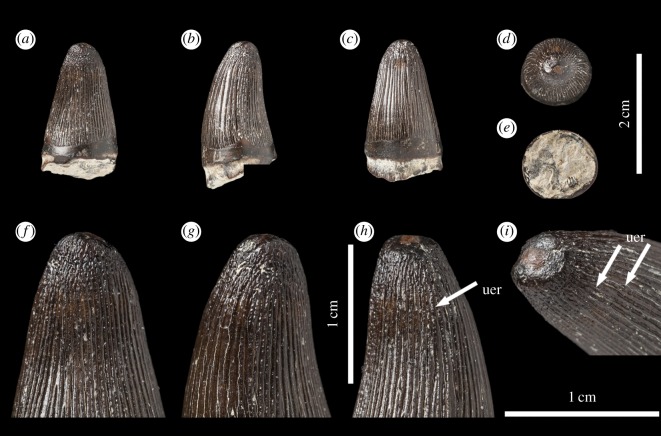
*Machimosaurus* sp., NHMUK PV R36793. Isolated tooth crown in (*a*) lingual view, (*b*) right lateral view, (*c*) labial view, (*d*) apical view, (*e*) basal view, (*f*) close-up on the apical-half in lingual view, (*g*) close-up on the apical half in lingual/right lateral view, (*h*) close-up on the apical-half in lingual/left lateral view, and (*i*) oblique close-up on the apex. uer, undulating apicobasal enamel ridge.

**Figure 2.**
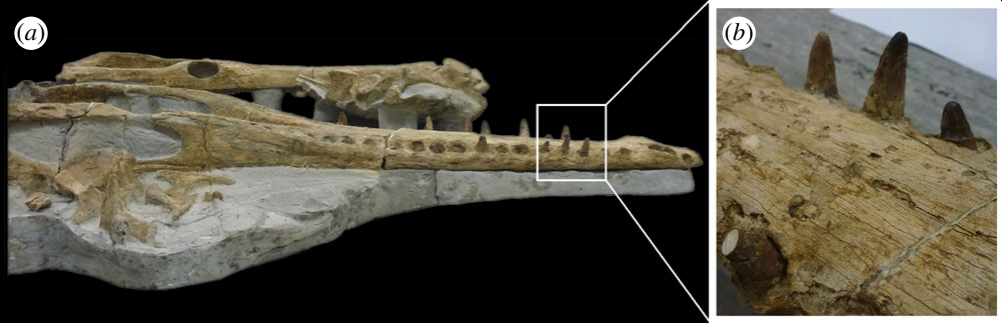
*Machimosaurus buffetauti*, SMNS 91415, holotype. (*a*) Lower jaw (with skull and associated post-crania) in right lateral view, and (*b*) close-up on the dentary dentition.

The taxonomically indeterminate *Machimosaurus* tooth is from Villerville, département du Calvados, Basse-Normandie, France (Calcaires gréseux d'Hennequeville Formation, upper Oxfordian, Upper Jurassic; figure 1). This specimen was first figured in Lepage *et al*. ([2, p. 98] and figure 2) alongside a second *Machimosaurus* tooth found in the same locality. These two teeth were originally registered under the number F14.VIV.19 in the private collection of Lepage *et al*. [2]. The specimen herein described is now registered as NHMUK PV R36793, while the second tooth is still within the Lepage collection.

The tooth is single cusped, conical, with little labiolingual compression, and is poorly lingually curved (figure 1). The apex is blunt and rounded, and shows evidence of being worn and polished. No apicobasal facets are evident on either the labial or lingual faces (such as those in the metriorhynchid genus *Geosaurus* [15,17]). Cingula (a ridge at the base of the crown) and accessory cusps/denticles are absent. The tooth also lacks carinae.

In addition to the blunt-apices, another characteristic feature of *Machimosaurus* dentition is the enamel ornamentation. Tooth enamel ornamentation varies along the crown; in the basal-mid regions, enamel ornamentation is composed of numerous apicobasally aligned ridges of high relief, which in the apical region shifts to an anastomosed pattern (figure 1). This general enamel ornamentation pattern is also seen in ‘*S.*’ *obtusidens* [1,13] and the metriorhynchid *To. carpenteri* [15,16] (these taxa also have blunt apices). Some of the apicobasally aligned enamel ridges, especially on the lingual surface, are ‘wavy’ or undulating. Where these undulations are very pronounced, they can be mistaken for serrations when seen without optical aids.

### 3.2 Tooth morphology of *Machimosaurus buffetauti*

**Figure 3.**
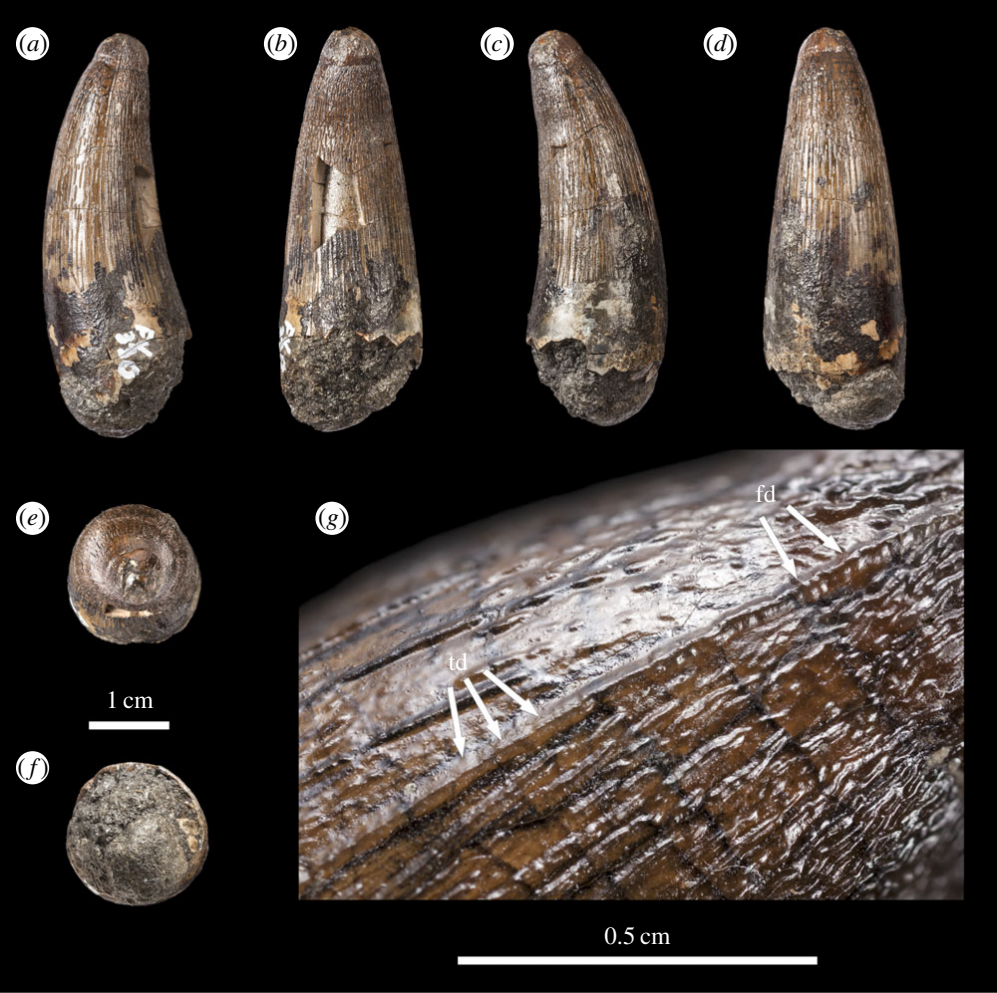
*Machimosaurus buffetauti*, DFMMh FV 330, referred specimen. Isolated tooth crown in (*a*) right lateral view, (*b*) lingual view, (*c*) left lateral view, (*d*) labial view, (*e*) apical view, (*f*) basal view, and (*g*) close-up on the right carinae. fd, false denticle; td, true denticle.

**Figure 4.**
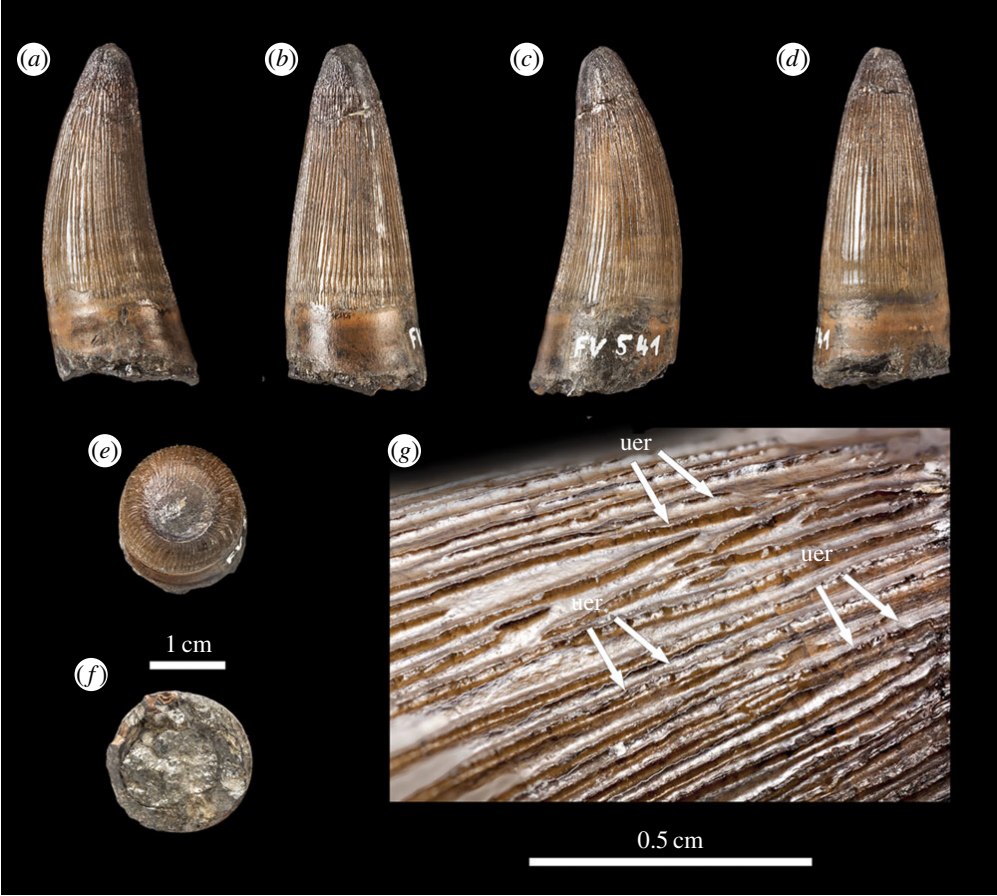
*Machimosaurus buffetauti*, DFMMh FV 541, referred specimen. Isolated tooth crown in (*a*) left lateral view, (*b*) lingual view, (*c*) right lateral view, (*d*) labial view, (*e*) apical view, (*f*) basal view, and (*g*) close-up on the apicobasal enamel ridges. uer, undulating apicobasal enamel ridge.

**Figure 5.**
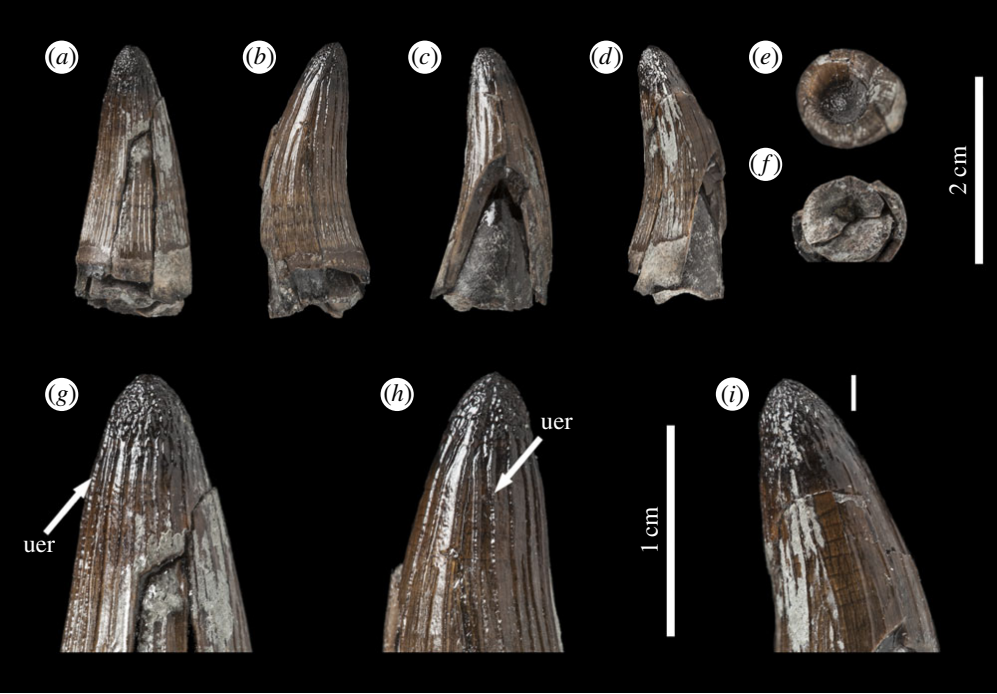
*Machimosaurus buffetauti*, MPV KIM027, referred specimen. Isolated tooth crown in (*a*) lingual view, (*b*) right lateral view, (*c*) labial view, (*d*) left lateral view, (*e*) apical view, (*f*) basal view, (*g*) close-up on the apical-half in lingual view, (*h*) close-up on the apical-half in right lateral view, and (*i*) close-up on the apical-half in left lateral view. uer, undulating apicobasal enamel ridge.

**Figure 6.**
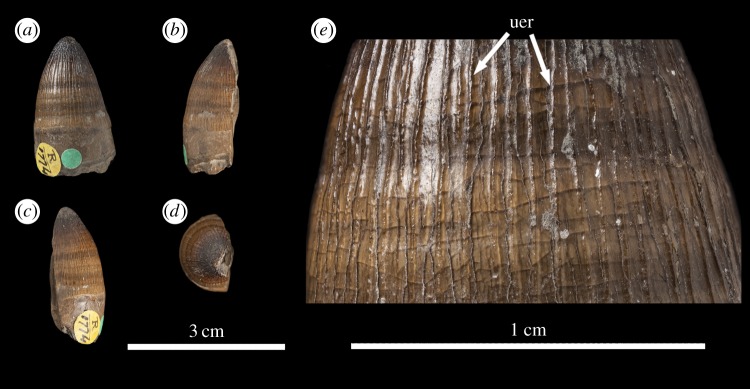
*Machimosaurus* cf. *buffetauti*, NHMUK PV R1774. Isolated tooth crown in (*a*) labial view, (*b*) right lateral view, (*c*) left lateral view, (*d*) apical view, and (*e*) close-up on the apicobasal enamel ridges. uer, undulating apicobasal enamel ridge.

The *Machimosaurus buffetauti* teeth described here are from Neuffen, Baden-Württemberg, Germany (Lacunosamergel Formation, lower Kimmeridgian, Upper Jurassic; figure 2), Langenberg near Oker, Lower Saxony, Germany (Langenberg Formation, Kimmeridgian; figures 3 and 4) and Cricqueboeuf, Normandy, France (Calcaires à *Harpagodes*Member of the Marnes de Bléville Formation, lower Kimmeridgian; figure 5). The *M*. cf. *buffetauti* tooth is from Smallmouth Sands, Dorset, UK (Kimmeridge Clay Formation, Kimmeridgian; figure 6).

The teeth of *M. buffetauti* are single cusped and conical, with little to no labiolingual compression (figures 2– 6). The apices are blunt and rounded, even in unworn crowns.

No apicobasal facets are evident on either the labial or lingual faces, and cingula and accessory cusps are absent. There is no evidence of carinal (mesiodistal) wear (such as in the metriorhynchid genus *Dakosaurus* [18]).

While the only *in situ* teeth in the holotype (SMNS 91415) are anterior-mid dentary teeth (figure 2), although there are some isolated crowns preserved in matrix, a referred specimen from Ain, France has *in situ* upper and lower jaw dentition [9]. This specimen shows heterodonty, with decreasing tooth apicobasal length along the tooth row. Isolated teeth referred to *M. buffetauti* from the Kimmeridgian of France and Germany (figures 3–5) [12], and cf. *M*. *buffetauti* from the Kimmeridgian of England (figure 6) [3] show the same variation in tooth apicobasal length. Interestingly, the anterior teeth of the holotype and the isolated, elongate teeth from France and Germany have carinae (figures 2–5), whereas the presumably posterior, shorter tooth from England lacks carinae (figure 6). This could suggest that presence of the carinae varies along the tooth row. Unfortunately, the *in situ* teeth of the Ain skull are still partially encased in matrix, so their carinae are not always visible [9]. The tooth crowns which are carinated have a mesial and a distal carina, and do not have split or supernumerary carinae (a carina that is split into multiple forks or multiple carinae located near each other [19]).

The tooth crowns are lingually curved, although this curvature is much more pronounced in the elongate tooth crowns (figures 2–5) than in the presumably posterior, shorter teeth (figure 6). Fortunately, the curvature of *in situ* Ain skull teeth is visible [9], and this specimen shows the same trend shown by the isolated tooth crowns: stronger lingual curvature of the elongate anterior teeth, and weaker lingual curvature of the shorter posterior teeth. Interestingly, some of the isolated tooth crowns preserved in matrix of the holotype (SMNS 91415) also show a shorter apicobasal length and are more poorly curved lingually than those *in situ* anterior-middle dentary teeth. Therefore, contra Martin & Vincent [6], the dentition is not homodont.

As with *Machimosaurus* sp., tooth enamel ornamentation varies along the crown. In the basal-mid regions, enamel ornamentation is composed of numerous apicobasally aligned ridges of high relief, which in the apical region shifts to an anastomosed pattern (figures 3–6). In *M. buffetauti* teeth, on the lingual tooth surface, the apicobasally aligned enamel ridges immediately adjacent to the apical anastomosed region are closely packed, whereas on the labial surface, these ridges are more widely spaced (figures 3–5). This is especially marked in the French tooth (figure 5). In the apical-half of the tooth crown, the apicobasally aligned enamel ridges are ‘wavy’ or undulating. Where these undulations are pronounced they closely resemble serrations (figures 3*g*, 4*g*, 5*g*,*h* and 6*e*).

Macrophotographic study of isolated *M. buffetauti* teeth shows true denticles on the carinae (figure 3*g*). These denticles are very similar to those seen on ‘*S*.’ *obtusidens*carinae [13], as they are microscopic, poorly defined and difficult to observe with the naked eye (table 1). They are present on both the mesial and distal carinae, but they do not proceed along the entire carina (heterogeneous), but rather appear in short rows. Note that some of the serrations in the apical region are, in fact, false denticles, created by the short anastomosed ridges contacting the carinae (figure 3*g* and table 2).

**Table 1.** List of characters accompanied by a description, to elucidate the various carinal/serration morphologies in Thalattosuchia. (This table is an updated version of table 1 presented in Young *et al*. [13].)

denticle size	denticles contiguous	denticle definition	description	examples
non-ziphodont	n.a.	n.a.	carinae present, but formed solely by a keel (raised ridge). No enamel ornamentation extending onto the carina, and no discrete denticles present	*Metriorhynchus superciliosus* *Gracilineustes leedsi*
macroziphodont	yes	well defined	carinae homogeneous, with a long, contiguous series of repetitive isolated and isomorphic true denticles that are visible macroscopically. Dimensions typically exceed 300 *μ*m	*Dakosaurus andiniensis* *Dakosaurus maximus*
microziphodont	yes	well defined	carinae homogeneous, with a long, contiguous series of repetitive isolated and isomorphic true denticles that are microscopic; whose dimensions typically do not exceed 300 μm	*Plesiosuchus manselii* *Geosaurus giganteus* *Geosaurus grandis* Geosaurinae indet. (NHMUK PV R486)
	yes	poorly defined and hard to discern	carinae homogeneous, with a long, contiguous series of repetitive isolated and isomorphic true denticles that are microscopic; whose dimensions typically do not exceed 300 μm. The denticles themselves are poorly defined and difficult to distinguish even under SEM (although this could be due to the enamel ornamentation extending onto the carina, especially in the apical region)	*Torvoneustes carpenteri*^*a*^
	no	poorly defined and hard to discern	carinae heterogeneous, with a series of non-contiguous repetitive isolated and isomorphic true denticles that are microscopic. Series can be short (2) or moderate (approx. 10), but are widely separate from one another, i.e. no contiguous series along the carina. Dimensions typically do not exceed 300 μm. In labial or lingual view, the height of the denticles rarely influences the height of the keel (i.e. little or no serrated edge). The denticles themselves are poorly defined and difficult to distinguish even under SEM	*Machimosaurus buffetauti*^*a*^ *Machimosaurus hugii*^*a*^ ‘*Steneosaurus*’ *obtusidens*^*a*^ ‘*Metriorhynchus*’ *brachyrhynchus* *Tyrannoneustes lythrodectikos*

^*a*^In these taxa the superficial enamel ornamentation extends onto the carina, especially in the apical region. In the absence of denticles this constitutes the false denticulated (false ziphodont) condition.

**Table 2.** List of serration and serration-like morphologies seen in the *Machimosaurus* teeth described and figured herein. (Note that determining true and false serrations along the carinae is hampered in most teeth as the carinae are either absent or are so poorly developed that they cannot easily be differentiated from the adjacent apicobasal enamel ridges.)

serration/serration-like morphologies	specimens which best show the morphology	species
enamel ridges contact the carinal keel (‘false ziphodonty’)	DFMMh FV 330	*Machimosaurus buffetauti*
	MG-unnumbered, NHMUK PV OR43638, NHMUK PV R232	*Machimosaurus hugii*
discrete units on the carinal keels (denticles)	DFMMh FV 330	*Machimosaurus buffetauti*
	NHMUK PV OR43638, NHMUK PV R5	*Machimosaurus hugii*
denticle-like discrete units on the enamel ridges	NHMUK PV OR33239, NHMUK PV OR43638, NHMUK PV R5, NHMUK PV R232	*Machimosaurushugii*
the apicobasally aligned enamel ridges are ‘wavy’/undulating (particularly on the lingual surface, and/or the apical half of the crown). Where these undulations are pronounced they mimic ‘denticle-like’ structures	NHMUK PV R36793	*Machimosaurus* sp.
	DFMMh FV 330, DFMMh FV 541, MPV KIM027, NHMUK PV R1774, SMNS 91415	*Machimosaurus buffetauti*
	NHMUK PV OR33239, NHMUK PV OR43638, NHMUK PV R5, NHMUK PV R232	*Machimosaurus hugii*

### 3.3 Tooth morphology of *Machimosaurus hugii*

The *M. hugii* teeth described here are from Solothurn, Canton Solothurn, Switzerland (Solothurn Turtle Limestone of the Reuchenette Formation, uppermost Kimmeridgian, Upper Jurassic; figures 7–15) and Guimarota, near Leiria, Portugal (Kimmeridgian, Upper Jurassic; figure 16).

**Figure 7.**
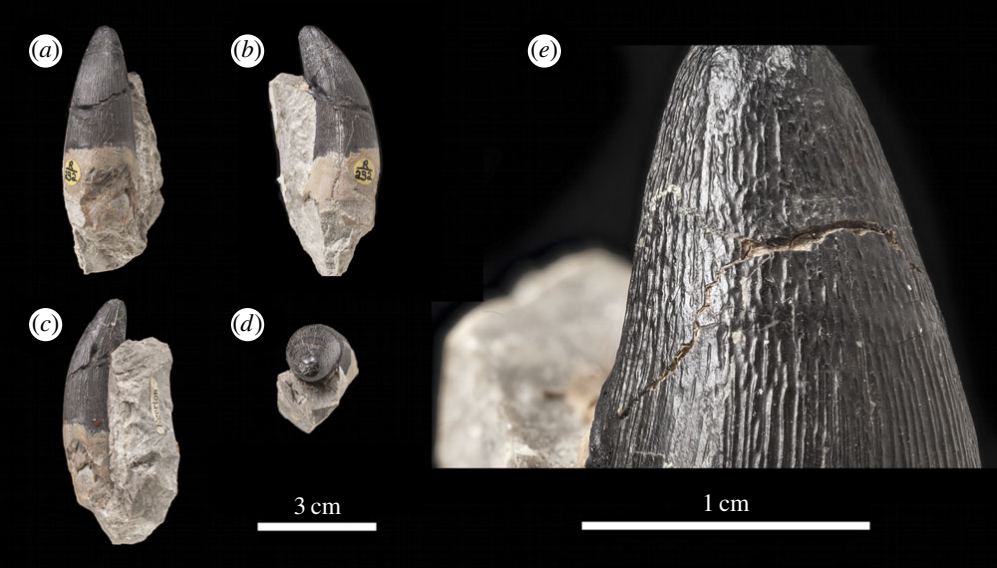
*Machimosaurus hugii*, NHMUK PV R232, referred specimen. Isolated tooth crown in (*a*) labial view, (*b*) left lateral view, (*c*) right lateral view, (*d*) apical view, and (*e*) close-up on the apicobasal enamel ridges.

**Figure 8.**
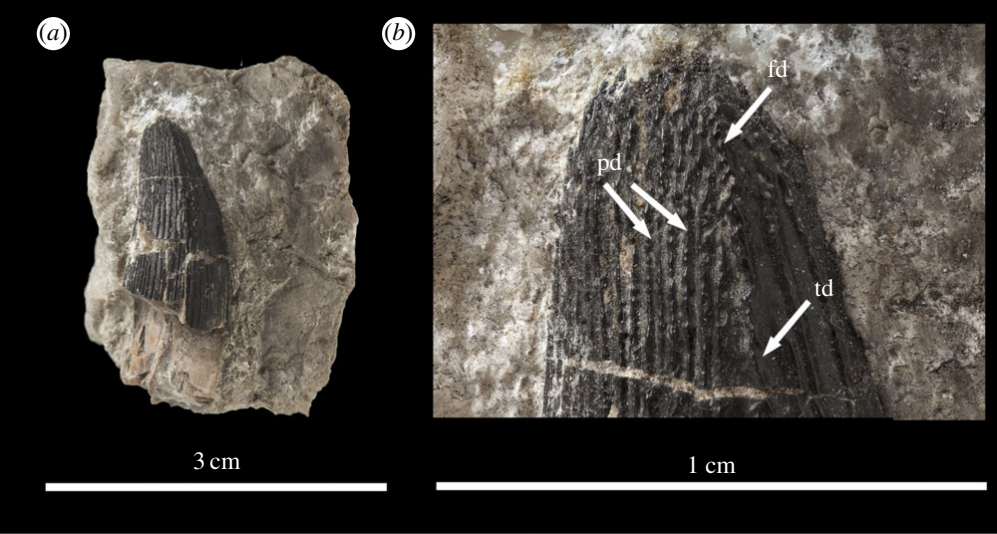
*Machimosaurus hugii*, NHMUK PV OR43638, referred specimen. Isolated tooth crowns in (*a*) left lateral view, and (*b*) close-up on the apicobasal enamel ridges. fd, false denticle; pd, ‘pseudo-denticle’ on an enamel ridge; td, true denticle.

**Figure 9.**
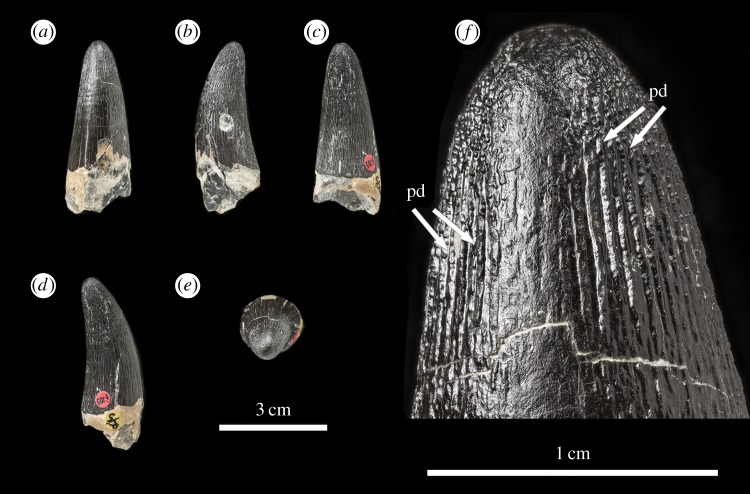
*Machimosaurus hugii*, NHMUK PV R5, referred specimen. First of the four isolated tooth crowns under that specimen number in (*a*) labial view, (*b*) right lateral view, (*c*) lingual view, (*d*) left lateral view, (*e*) apical view, and (*f*) close-up on the apicobasal enamel ridges. pd, ‘pseudo-denticle’ on an enamel ridge.

**Figure 10.**
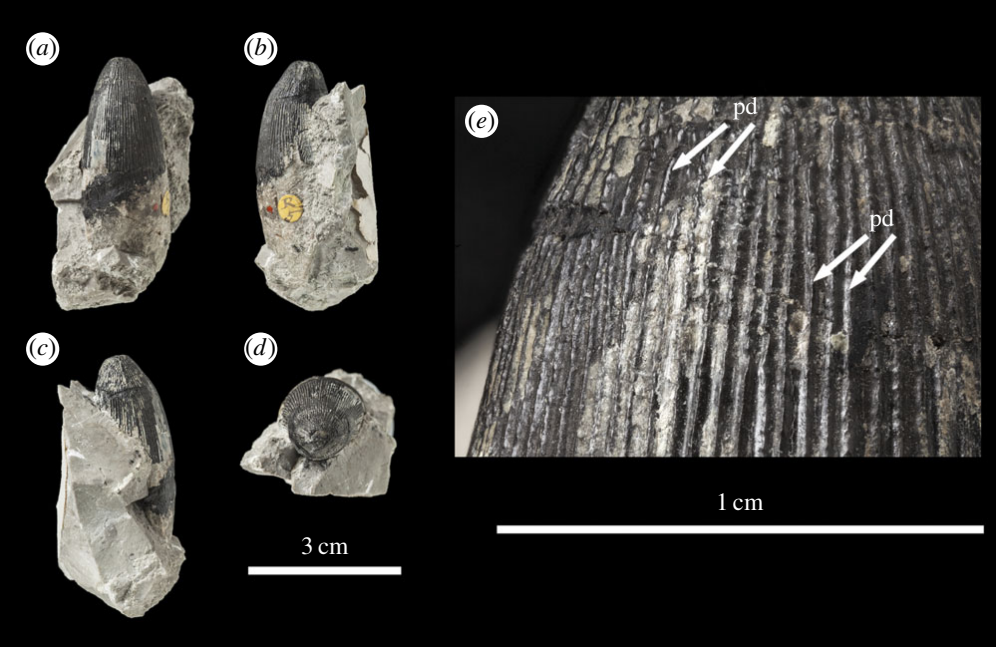
*Machimosaurus hugii*, NHMUK PV R5, referred specimen. Second of the four isolated tooth crowns under that specimen number in (*a*) labial view, (*b*) right lateral view, (*c*) left lateral view, (*d*) apical view, and (*e*) close-up on the apicobasal enamel ridges. pd, ‘pseudo-denticle’ on an enamel ridge.

**Figure 11.**
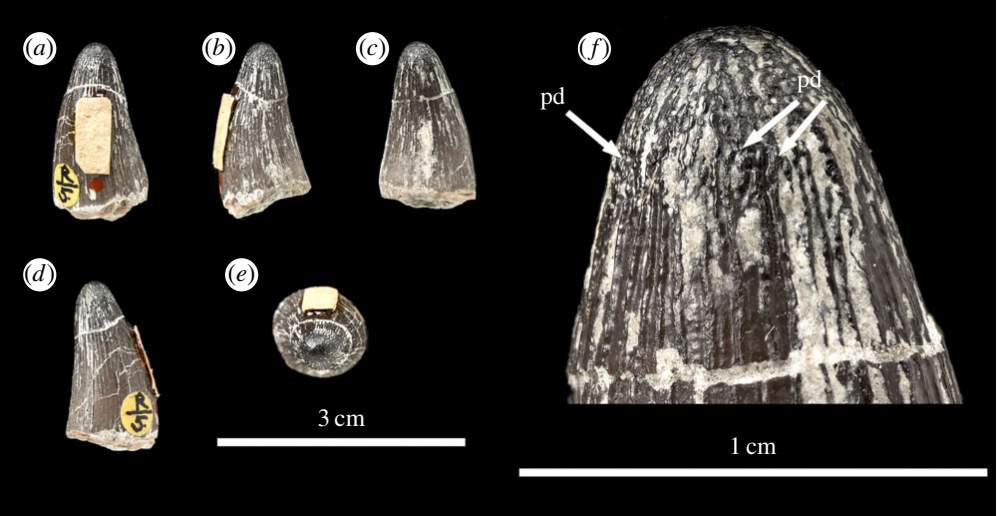
*Machimosaurus hugii*, NHMUK PV R5, referred specimen. Third of the four isolated tooth crowns under that specimen number in (*a*) labial view, (*b*) left lateral view, (*c*) lingual view, (*d*) right lateral view, (*e*) apical view, and (*f*) close-up on the apicobasal enamel ridges. pd, ‘pseudo-denticle’ on an enamel ridge.

**Figure 12.**
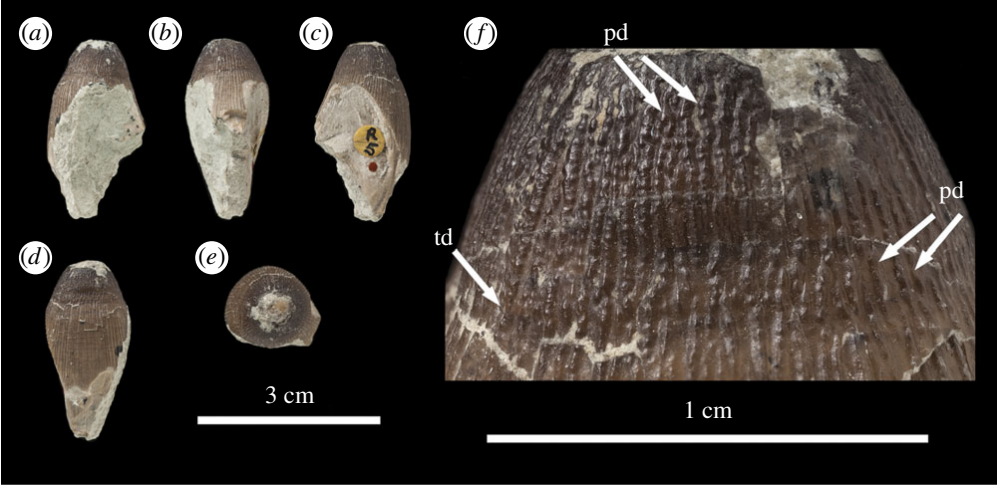
*Machimosaurus hugii*, NHMUK PV R5, referred specimen. Fourth of the four isolated tooth crowns under that specimen number in (*a*) right lateral view, (*b*) lingual view, (*c*) left lateral view, (*d*) labial view, (*e*) apical view, and (*f*) close-up on the apicobasal enamel ridges. pd, ‘pseudo-denticle’ on an enamel ridge; td, true denticle.

**Figure 13.**
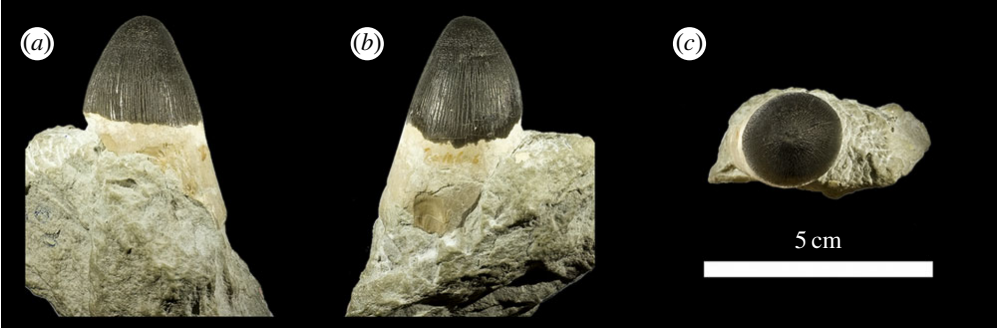
*Machimosaurus hugii*, NMS 8342, lectotype. Isolated tooth crown in (*a*) labial view, (*b*) lingual view, and (*c*) apical view.

**Figure 14.**
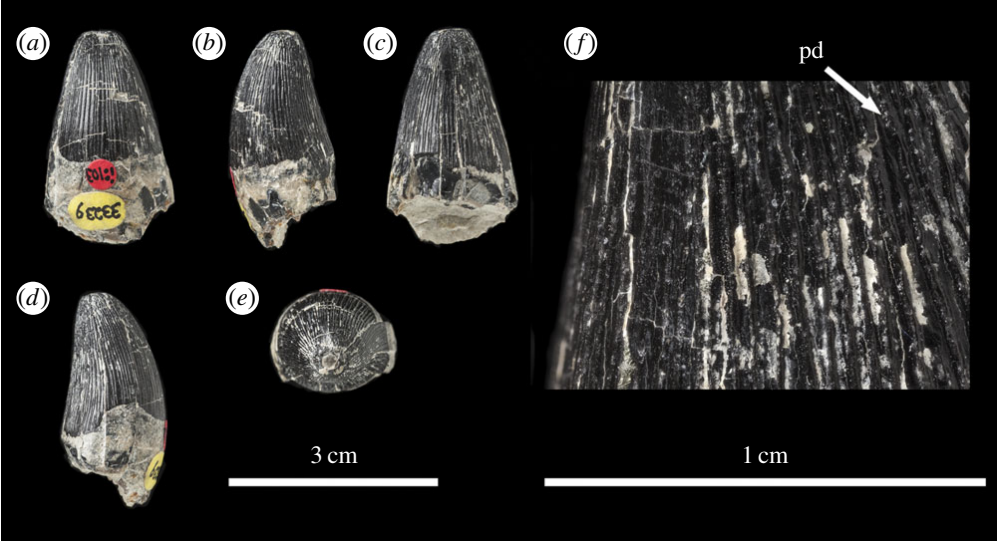
*Machimosaurus hugii*, NHMUK PV OR33239, referred specimen. First of the two isolated tooth crowns under that specimen number in (*a*) labial view, (*b*) right lateral view, (*c*) lingual view, (*d*) left lateral view, (*e*) apical view, and (*f*) close-up on the apicobasal enamel ridges. pd, ‘pseudo-denticle’ on an enamel ridge.

**Figure 15.**
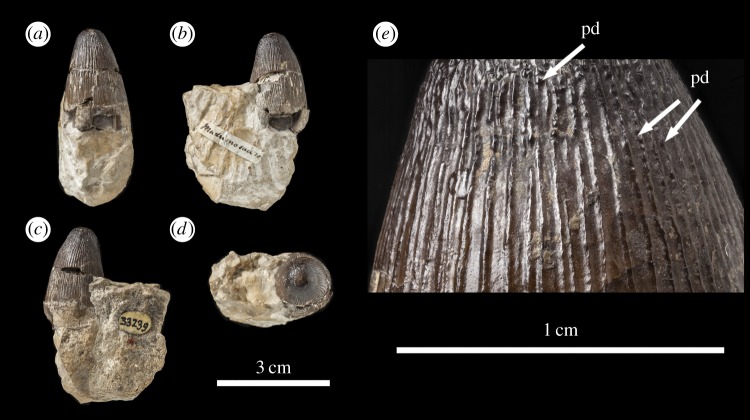
*Machimosaurus hugii*, NHMUK PV OR33239, referred specimen. Second of the two isolated tooth crowns under that specimen number in (*a*) labial view, (*b*) left lateral view, (*c*) right lateral view, (*d*) apical view, and (*e*) close-up on the apicobasal enamel ridges. pd, ‘pseudo-denticle’ on an enamel ridge.

**Figure 16.**
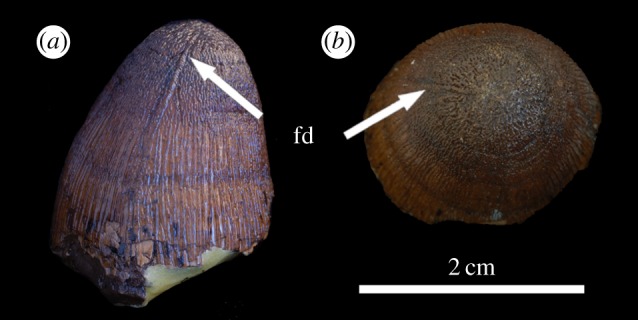
*Machimosaurus hugii*, MG unnumbered, referred specimen. Isolated tooth crown in, (*a*) right lateral view, and (*b*) apical view. fd, false denticle.

The teeth of *M. hugii* are single cusped and conical, with little to no labiolingual compression (figures 7–16). The apices are blunt and rounded, even in unworn crowns. No apicobasal facets are evident on either the labial or lingual faces, there is no evidence of carinal (mesiodistal) wear, and cingula and accessory cusps are absent.

Unfortunately, there are no *M. hugii* specimens which show the variation across *in situ* upper and lower jaw tooth rows. However, the variation of isolated *M. hugii* teeth is identical to that seen in *M. buffetauti* (variation in apicobasal length and lingual curvature, and presence/absence of the carinae), which is suggestive of heterodonty. There are tooth crowns which are elongate, have a pronounced lingual curvature and are carinated (figures 7–9); ones which are shorter, have a less pronounced lingual curvature and are carinated (figures 10–12); and some that are even shorter (subglobidont) teeth, which are poorly curved and are either uncarinated or the carinae are restricted to the apical region (figures 13–16). The tooth crowns which are carinated have a mesial and a distal carina, and do not have split or supernumerary carinae.

As with *M. buffetauti*, the tooth enamel ornamentation varies along the crown. In the basal-mid regions, enamel ornamentation is composed of numerous apicobasally aligned ridges of high relief, which in the apical region shifts to an anastomosed pattern (figures 7–16). Again, as with *M. buffetauti*, there is variation in the ornamentation patterns on the labial and lingual surfaces. On the lingual tooth surface, the apicobasally aligned enamel ridges immediately adjacent to the apical anastomosed region are closely packed, whereas on the labial surface these ridges are more widely spaced (figures 7–9). However, this only occurs on the elongate tooth crowns. In the shorter tooth crowns, the enamel ornamentation patterns of the labial and lingual surfaces are identical (figures 10–16). In the apical half of the tooth crown, the apicobasally aligned enamel ridges are ‘wavy’ or undulating, but these undulations are notably less pronounced than those seen in *M. buffetauti*.

Macrophotographic study of isolated *M. hugii* teeth shows true denticles on the carinae (figures 8*b*, 11*f*, 12*f* and 16*a*). These denticles are very similar to those of *M. buffetauti* and ‘*S*.’ *obtusidens* [13], in that they are microscopic, poorly defined, and are difficult to observe (table 1). They are present on both the mesial and distal carinae, but they do not proceed along the entire carina (heterogeneous), but rather appear in short rows. As with *M*. *buffetauti*, some of the serrations in the apical region are in fact false serrations, created by the short anastomosed ridges contacting the carinae (figures 8*b* and 16*a*).

One of the most curious features of the isolated *M*. *hugii* teeth is seen along the apicobasal and anastomosed ridges. On these ridges are what look like accessory denticles (best seen in figures 8–15). These structures are more numerous, larger and better defined than the true denticles and false serrations seen on the carinae, such that they can be seen with the naked eye, without optical aids, in good lighting. These ‘pseudo-denticles’ are restricted to the enamel ridges on the apical half of the tooth crowns.

## 4. Discussion

### 4.1 Co-occurrence of true denticles and false denticles

The co-occurrence of both true denticles and false denticles (superficial enamel contacting the carinal keel) on the carinae of *M. buffetauti* and *M. hugii* teeth is notable, but not unique to these species. These two serration morphologies also co-occur in the teleosaurid ‘*S.*’ *obtusidens* and the metriorhynchid *To. carpenteri* [15,16], and possibly also in the ‘pholidosaurid’/elosuchid *Elosuchus cherifiensis* ([20], MNHN.F MRS 340 and other currently undescribed MNHN *Elosuchus* specimens). All of these taxa share: robust conical teeth, blunt apices and an enamel ornamentation pattern that in the apical region transitions from being apicobasally aligned to an anastomosed pattern. It is in this anastomosed region that the enamel ridges contact the carinal keel and forms false denticles. It has been hypothesized that the co-occurrence of these morphologies may be linked to functional ecology and diet, specifically a durophagous/generalized macrophagous diet in which both crushing and flesh-slicing are important [13]. It is an interesting hypothesis, which study on durophagous taxa from other clades (such as mosasaurids and other crocodylomorph clades) could help test. Regardless of the precise reason why true denticles and false denticles occur together, the fact that they are found together in a small group of teleosaurids and metriorhynchids suggests that these animals (*Machimosaurus* spp., ‘*S.*’ *obtusidens*, *Torvoneustes*) had a generally similar diet, and may have filled somewhat similar roles in their respective ecosystems.

Apart from the co-occurrence of true and false denticles, most of these characteristics (heterodont dentition, the posterior-most teeth having a robust, conical shape with blunt apices, false denticles and an enamel ornamentation pattern that in the apical region transitions from being apicobasally aligned to an anastomosed pattern) are found in other marine crocodylomorphs. These include, most notably, the dyrosaurid *Phosphatosaurus* *gavialoides* [12] (and cf. *Phosphatosaurus* MNHN.F APH 23) and the tomistomine crocodylian *Maroccosuchus zennaroi* ([21], MNHN.F APH 18). As such, this dentition type evolved repeatedly during crocodylomorph evolution.

### 4.2 Pseudo-denticles

Perhaps the single most striking feature of any of the *Machimosaurus* teeth described here is the ‘pseudo-denticles’ on the apicobasal and apical anastomosed ridges of *M. hugii* crowns. Their presence helps to readily differentiate the teeth of this taxon from other blunt-toothed teleosaurids (such as ‘*S.*’ *obtusidens*, *M. buffetauti* and *Machimosaurus* sp.). These are large, well-defined and immediately notable structures, but their potential function is unclear. The ‘pseudo-denticles’ increase the surface area of the apical region of the tooth crowns. This could be a way of maximizing friction, and therefore facilitating grip, on wet prey items (such as marine turtles, some of which have embedded *M. hugii* teeth and/or bite marks consistent with *M. hugii* [4]). These structures could have also have strengthened the enamel.

It is interesting to note that many other durophagous vertebrates have structures that are broadly similar to the ‘pseudo-denticles’ of *M. hugii*, although usually not as large and well defined. These include, for example, various mosasaurids [22], bony fishes such as *Paralbula* [23], crocodylomorphs such as *Brachychampsa* [24,25], and some mammals (such as the ‘condylarth’ *Periptychus* [26]). All of these taxa possess complex and often anastomosing enamel ridges that undulate, sometimes with bulbous projections like the ‘pseudo-denticles’. A broader comparative study or biomechanical study is beyond the scope of this paper, but it is possible that these similar structures served to increase surface area for crushing, strengthened the tooth enamel to better withstand hard-objecting feeding, or both (as has been hypothesized for durophagous mosasaurids [27]). There is some support for the increased friction hypothesis from testing on shoe tread–groove design, which found that tread design influences friction, especially in the presence of liquids [28]. However, relating these tread–groove results to tooth crown ‘pseudo-denticles’ and the ‘interpseudo-denticular sulci’ would require biomechanical testing.

## 5. Conclusion

Here, we comprehensively describe and figure the dentition of two of the most distinctive species of Late Jurassic marine crocodylomorphs, the teleosaurids *M. buffetauti* and *M. hugii* (and described an isolated *Machimosaurus* sp. tooth). We find that both species had heterodont dentition with a variety of enamel ornamentation patterns, including four distinct types of serrations or serration-like structures. These findings therefore increase the roster of serrated taxa within Teleosauridae, a group that was long thought to lack tooth serrations. *Machimosaurus buffetauti* and *M*. *hugii* share the same suite of morphologies as the closely related species ‘*S.*’ *obtusidens*. All these taxa have true denticles and false denticles co-occurring on the same teeth. As such, there is now a subclade of teleosaurids which have: proportionally enlarged supratemporal fenestrae, foreshortened rostra, reduced tooth counts, teeth with blunt apices and heavily ornamented dentition (apicobasally aligned ridges in the basal-mid regions and shorter anastomosed ridges apically) with serrated carinae. The discovery of serrated dentition in *Machimosaurus* strengthens the contention that the ‘*S*.’ *obtusidens*–*Machimosaurus* subclade may have had a diet broader than simple durophagy, with flesh slicing also part of their feeding repertoire.

The most curious discovery is the presence of ‘pseudo-denticles’ on the enamel ridges of *M. hugii* teeth. This morphology has never been described for a marine crocodylomorph to the best of our knowledge. These ‘pseudo-denticles’ are better defined and more numerous than the true denticles along the carinae. The presence of this morphology could be a way to increase the surface area of the apical region of the crowns for maximizing friction (which would be advantageous for maximizing grip), or may have strengthened the tooth enamel. However, these hypotheses require biomechanical testing. Nevertheless, the presence of this peculiar morphology demonstrates that thalattosuchian dentition still yields surprises, despite the long-held assumption of dental conservatism in this group.
